# Practices for promoting a child’s best interests in paediatric rehabilitation – Perspectives of professionals and parents

**DOI:** 10.1177/13674935241287880

**Published:** 2024-09-28

**Authors:** Nea Vänskä, Salla Sipari, Leena Haataja

**Affiliations:** 1 Future Proof Health and Wellbeing Innovation Hub, 52907 Metropolia University of Applied Sciences, Helsinki, Finland; 2 Department of Pediatric Neurology, 60655 University of Helsinki, Helsinki, Finland; 3 Children´s Hospital and Pediatric Research Center, 3836 Helsinki University Hospital, Helsinki, Finland

**Keywords:** Child’s best interests, children with disabilities, collaboration, patient participation, paediatric rehabilitation

## Abstract

Practices for promoting a child’s best interests in rehabilitation are not sufficiently understood. This study describes the practices from the perspectives of professionals and parents of children with disabilities. We conducted 11 interviews: 5 in focus groups with professionals (*n* = 27 [69%]), 3 with parents (*n* = 9 [23%]), and 3 individual interviews of paediatric neurologists (*n* = 3 [8%]). We used a qualitative approach, which included inductive content analysis, to examine the transcribed interview data. The practices for promoting a child’s best interests consisted of collective framing of child-specific rehabilitation, fostering a fulfilling daily life for the child, and ensuring appropriate rehabilitation. This was enhanced by using child-specific practices and comprehensively understanding the child’s rehabilitation in everyday life but was hindered by the absence of an established process and guidelines. The results highlighted substantial challenges in collaboration aligned with the child’s best interests, enabling the child’s active participation, and addressing the individual needs of the child and family. Promoting best interests through family–professional partnerships by using a systemic and ecological approach could guide the rehabilitation process and ensure the child’s right to participate.

## Introduction

Every child has the right to participate and to have their best interests assessed and taken into account as a primary consideration in all decision-making concerning them (Committee on the Rights of the Children, 2013; United Nations, 2006). The child’s best interests must be assessed on a case-by-case basis, considering the specific circumstances, individual characteristics and needs, and the short- and long-term consequences for a group of children or a specific child (Committee on the Rights of the Children, 2013; Quaye et al., 2021; Ruggiero, 2022).

The child’s best interests principle’s purpose is to secure a child’s overall development and to ensure realisation of their rights (Committee on the Rights of the Children, 2013), that is, safety, healthcare, education, family relationships, physical and psychological wellbeing, emotional growth, identity, participation, privacy, and their agency to form and express their views (Information Commissioner’s Office, 2020; Streuli et al., 2011). In practice, acting in the child’s best interests includes generating an understanding of what is best for a child in a certain situation (Birchley, 2014; Streuli et al., 2021). This involves evaluating and balancing relevant factors in the context and in services comprehensively, including the child’s perspective and diverse perspectives of related stakeholders (General Medical Council, 2018; Paul, 2007; Streuli et al., 2011). Thus, implementing the child’s best interests in practice requires individual and collaborative adaptation in each specific context (Ruggiero, 2022), such as in paediatric rehabilitation.

Children’s best interests are highly relevant in rehabilitation (Foster et al., 2022; Sipari et al., 2017; Streuli et al., 2011), where they are promoted through family–professional collaboration (Birchley et al., 2022; Streuli et al., 2011). Due to the complex health needs of children with disabilities, rehabilitation is a long-term, interactive process and includes multidisciplinary collaboration with professionals across organisational boundaries (Järvikoski, 2013; Rosenbaum and Gorter, 2012; Trabacca et al., 2016). Consequently, ensuring child’s best interests in practice can be challenging and can involve multiple competing interests and pressures (Birchley et al., 2022).

The child’s participation in family–professional collaboration with shared decision-making is a pivotal factor in successful rehabilitation (Järvikoski et al., 2015; McCoy et al., 2020). However, previous research demonstrates a considerable lack of knowledge on how to promote a child’s participation in their rehabilitation process (Foster et al., 2022; Teleman et al., 2021), and parents’ and professionals’ actions strongly influence a child’s opportunities to participate (Coyne and Harder 2011; Quaye et al., 2021; Teleman et al., 2021). A lack of suitable opportunities, resources, and support hampers children’s and young people’s developing capacity to take care of their health, engage in their communities, and exercise their citizenship (Gorter and Gibson, 2015). Although participation- and evidence-based practices underline collaboration (Anaby et al., 2018; Palisano et al., 2012) and identification of a child’s perspectives and child-determined goals (Costa et al., 2017), rehabilitation services continue to be criticised for being professional-led (Piškur et al., 2016), adult-centred (Teleman et al., 2021), and not focussing on realising children’s agency (Bekken, 2014; Coyne et al., 2016).

Although the literature demonstrates dilemmas in acting in the child’s best interest (Coyne and Harder 2011) and complexity of ensuring a child’s best interests in family–professional collaboration (Birchley et al., 2022; Woo et al., 2015), studies of promoting a child’s best interests in rehabilitation have remained scarce and practice guidelines are lacking. Current knowledge on promoting child’s best interests is focused on, for example, decision-making in life-threatening situations such as intensive medical care (Birchley, 2014), and on realising a child’s participation rights in an institutional context such as a hospital (Foster et al., 2022; Quaye et al., 2021): we still know little about how the children’s best interests are promoted in the multidisciplinary rehabilitation process (März, 2022). To date, no study has looked specifically at current and required practices for promoting a child’s best interests in paediatric rehabilitation, and this study aims to shed light on this knowledge gap.

## Aim

To explore how professionals and parents of children with disabilities perceive the practices for promoting a child’s best interests in paediatric rehabilitation.

## Method

### Study design and population

We used a qualitative research design in this study. We conducted focus group and individual interviews to obtain parents’ and professionals’ perspectives to better understand the study phenomenon (Barbour, 2007). According to the constructivism paradigm, knowledge is constructed through social interaction (Guba and Lincoln, 1994), and thus the focus group discussions enabled exploring participants’ multiple perspectives through dialogue (Barbour, 2007). The individual interviews enabled knowledge production by individuals who were not able to join the group sessions.

**Figure 1.**
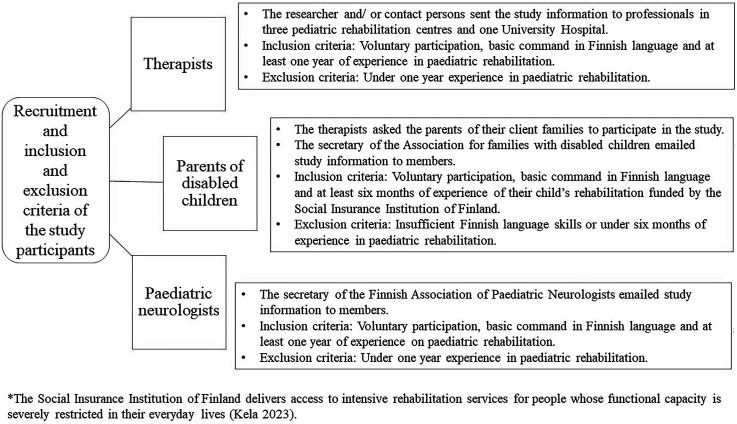
Participant recruitment with inclusion and exclusion criteria. *(Kela, 2023)

This study’s population consisted of parents and paediatric rehabilitation professionals from Finland, where multidisciplinary rehabilitation is planned in public sector and often realised by professionals in private rehabilitation centres. Thus, we recruited rehabilitation experts from both public and private sectors: therapists who provide paediatric rehabilitation, parents, and paediatric neurologists. Figure 1 illustrates the recruitment process with purposive sampling and inclusion and exclusion criteria.

A study approval from participating organisations and Ethics Committee of Universities of Applied Sciences in Helsinki Metropolitan area was received as part of the LOOK-project (https://look.metropolia.fi/in-english/). We asked participants to sign an informed consent form after they received oral and written information.

### Data collection

The interviews emphasised participants’ perspectives on current and required practices for promoting a child’s best interests in rehabilitation. The researcher facilitated the discussion by using flexible, open-ended questions based on the interview themes, which were formulated from the literature (Table 1). Follow-up questions aimed to help participants elaborate and clarify their statements. The audio-recorded interviews were conducted by NV, transcribed verbatim by research assistant, and pseudonymised (Table 2) by NV.

**Table 1.** Interview themes, example questions, and prompts for discussion.

Interview themes	Examples of questions
Main theme: Practices for promoting a child’s best interest in rehabilitation	
1) Child’s agency and participation	How is a child’s active participation and influence over their own matters enabled in rehabilitation?
	Can you give a concrete example of a practice that enables a child’s participation?
2) Child, family, and professional collaboration	How is a child’s participation in the collaboration with adults enabled in rehabilitation?
	What collaboration is needed in rehabilitation according to the child’s best interests?
3) Realisation of child’s best interests in practice	Can you describe your perspectives on what needs to be taken into account when making decisions on the child’s best interests in rehabilitation?
	How are the child’s best interests promoted in rehabilitation?
	Can you give an example of a practice for promoting the child’s best interests in rehabilitation?

**Table 2.** Example of meaning units, condensed meaning units, codes, and subcategory.

Meaning unit	Condensed meaning unit	Code	Subcategory
‘You can pay attention to even a small child’s thoughts about therapy’ (Eve, therapist)	Pay attention to child’s thoughts about therapy	Exploring child’s views	Exploring and including child’s perspectives
‘You can ask the child to assess what benefits they have noticed from therapy’ (Kaisa, paediatric neurologist)	Ask child to assess therapy benefits		
‘…I prefer to interview and talk with the child about things in their daily life’ (Tanja, paediatric neurologist)	Interviews and discussion with child about daily life		
‘The things that the child brought up in the discussion was taken seriously. We really took them into consideration and there were many of us in the meeting’ (Krista, therapist)	Things that were important to child were considered in meeting	Taking child’s perspectives into account	

### Data analysis

We outlined a research question based on the study aim: ‘What are the current and required practices for promoting a child’s best interests in rehabilitation from the perspectives of professionals and parents of children with disabilities?’. Inductive content analysis (Elo and Kyngäs, 2008; Graneheim and Lundman 2004) was used for data analysis. First, the transcripts were read. Then the transcripts’ meaning units that comprised words, phrases, or sentences answering to study question were identified. The analysis proceeded to condensing the meaning units and shortening them into codes without losing their original core meaning. Analysis continued into grouping the codes according to their similarities. Next, the codes were sorted into subcategories using abstraction (Table 2).

The analysis proceeded to identify similarities and differences between the subcategories, which were further abstracted into categories. The logic of the analysis was checked in relation to its steps to ensure consistency. Finally, main categories were formulated to describe the practices for promoting a child’s best interests. The analysis was conducted by NV. To reinforce trustworthiness, throughout the process the authors systematically reflected on the transcribed interviews, codes, categories, and results.

## Findings

The data consisted of 11 interviews that lasted 79–129 min (mean time 106.7 min): 5 focus group interviews of rehabilitation professionals (*n* = 27), 3 focus group interviews of parents (*n* = 9), and 3 individual interviews of paediatric neurologists (*n* = 3). Three focus groups had two participants because of last-minute withdrawals. Due to difficulties participating in group discussions at fixed times, three individual interviews were carried out with paediatric neurologists, who often play a central role in determining a child’s best interests. The interviews are described in Supplemental Material 1. Table 3 describes the participants.

**Table 3.** Study participants.

Participants *N* = 39 (100%)	Occupation	Experience in rehabilitation (median/IQR)	Work sector
Professionals *n* = 30 (77%)	Paediatric neurologists *n* = 7 (23%)	20 years/6	Hospital/university hospital *n* = 2 (29%) Provider of access to rehabilitation *n* = 1 (14%) Disability outpatient clinic *n* = 3 (43%) Centre for learning and consulting *n* = 1 (14%)
	Physiotherapists *n* = 11 (37%)	27 years/24	Rehabilitation centre *n* = 8 (73%) University hospital *n* = 3 (27%)
	Occupational therapists *n* = 9 (30%)	13 years/6	Rehabilitation centre *n* = 6 (67%) University hospital *n* = 3 (33%)
	Speech therapists *n* = 3 (10%)	9 years/22	University hospital *n* = 3 (100%)
Parents *n* = 9 (23%)	Mother/ father	Children’s disabilities *N* = 9	Children’s ages (median/IQR)
	Mothers *n* = 8 (89%) Father *n* = 1 (11%)	Visual impairment and hemiplegia *n* = 1 (11%) Cerebral palsy *n* = 5 (56%) Autism *n* = 2 (22%) Progressive neurological disorder *n* = 1 (11%)	9 years/3,5

**Figure 2.**
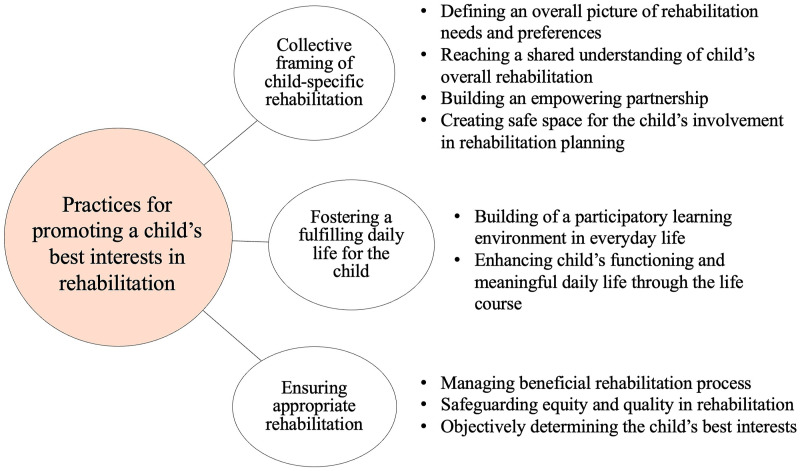
Practices for promoting a child’s best interests in rehabilitation from parents’ and professionals’ perspectives.

Through the analysis, 46 subcategories, 9 categories, and 3 main categories answering to study question were identified. Supplemental Material 2 presents a full table of the results. The three main categories describing the practices for promoting a child’s best interests in rehabilitation were (1) collective framing of child-specific rehabilitation, (2) fostering a fulfilling daily life for the child, and (3) ensuring appropriate rehabilitation. Figure 2 illustrates the findings.

### Collective framing of child-specific rehabilitation

By *defining an overall picture of rehabilitation needs and preferences*, the amount and quality of a child’s rehabilitation were individually assessed and outlined.

‘The kind of rehabilitation required needs to be considered and applied individually, it’s not one and the same package for everyone. This is why we have to assess it so carefully, have many pairs of eyes check it, discuss issues, and collect multifaceted information…’ (Heljä, Paediatric Neurologist).

Professionals explained that the child’s functioning, everyday needs, and developmental trajectories were assessed to draw up a rehabilitation plan. They indicated that a child’s emotional and physical wellbeing, learning difficulties, social relationships, risk of social exclusion, and problems in everyday life needed to be identified and examined if concerns arose.

Combining experience- and evidence-based knowledge and considering diverse views was essential for identifying key rehabilitation issues. The importance of exploring parents’ perspectives was highlighted by all. Exploring and including the child’s perspective was necessary for identifying their needs and preferences.

‘Well, if you really want to imagine what would be in a child’s best interests then yes, you should ask for the child’s view, of course. What’s important to them. And then ask for the parents’ views. -- Then also other views to find out things you didn’t know. To really see what issues are important’ (Minna, Parent).

A shared understanding of the child’s overall rehabilitation was created by balancing perspectives through negotiation between family members and professionals. Joint planning aimed to create a consensus on what is best for the child and was considered important by most participants.

‘It’s in the best interests of the child that parents and professionals have a shared understanding, and that we are all looking in the same direction. We need to avoid conflicts in these issues – that’s the worst-case scenario. Perspectives can be a little different, but that’s ok; then we discuss them’ (Tanja, Paediatric Neurologist).

Collaborative deliberation was perceived challenging by many, and they questioned how conflicting views and priorities should be handled. Creating a shared understanding was challenged by, for example, the number of people and perspectives involved, disagreements within the family, and cultural differences.

‘It’s hard to think (what is best for a child), because you have to consider the child's coping and what the child wants, and then when everyone has their own opinions’ (Hanna, Parent).

Participants stressed a need for a comprehensive and unified plan and goals in the child’s rehabilitation network, rather than separate plans and isolated, overlapping goals from various organisations, to ensure that rehabilitation aligns with the child’s best interests. A comprehensive rehabilitation plan would consider the family’s resources, timetables, and strengths and help coordinate families’ everyday demands and rehabilitation.

Katariina ‘It’s annoying that every therapist working with a child and their family establishes three to four goals and no one discusses what we’re doing’.

Pinja ‘Yes, establishing these goals together is challenging… One family might have over 12 goals...’ (Therapists’ discussion).

Participants perceived *building an empowering partnership* vital. Recognising the central role parents play in rehabilitation, supporting their active participation and commitment was deemed crucial. Practices like pre-meeting preparation, collaborative needs-analysis, and problem-solving facilitated parents’ involvement as well as professionals’ clear communication. Participants emphasised the importance of acknowledging parents’ knowledge and counselling needs. Parents sought assurance that their child received optimal care and rehabilitation, while also needing reassurance about their efforts for their child’s wellbeing. Parents valued professionals’ emotional support in decision-making, trustworthy and specific information, and concrete guidance in everyday life situations. Arranging parental support for daily tasks and coping were essential, but in current practice, psychological and concrete support for parents was scarce.

‘For example, you really need concrete guidance on how to deal with a baby and how to assist, turn and lift her when she gets bigger. -- everyday guidance like that is the most important’ (Leila, Parent).

Trust and continuance in the family–professional relationship was important and, at best, professionals and parents learned together how to best support the child’s overall development. Parents appreciated a good relationship with the professionals, emphasising respect for the family’s cultural background and privacy. Promoting a child’s best interests encompassed empowering both child and family. Emphasising opportunities, learning, overall health, and wellbeing, rather than focussing solely on disability, as well as professionals’ assistance in individual adjustment processes, supported family empowerment. Fostering strong family ties, positive relationships, and enjoyable family activities were deemed important. Parents appreciated therapists aiding in envisioning long-term goals and empowering visions for the family and child’s future.

‘Hanna: We could really think that families with disabled children are superfamilies. Linda: Yes, indeed. Because it doesn’t only affect the child’ (Parents’ discussion).

Promoting a child’s best interests included supporting the child’s positive and realistic self-perception through focussing on strengths and solutions, encountering the child as a child, not as a diagnosis, and respecting the child’s personality. Trust in the child’s potential, fostering their belief in coping, and enabling the child to practice and succeed in real-life tasks helped build their self-reliance. The child learning to recognise their needs and decide when to seek help or handle tasks independently was important.

*A safe space for the child’s involvement* in rehabilitation planning was created through individual support and sensitive interaction using the child’s communication methods, respecting their privacy and acknowledging their emotions. Using visual and adaptive methods and tools such as pictures and photos helped children to express themselves. Enabling child involvement included pre-preparation, a trusting relationship, unhurried discussions, and clear information about rehabilitation. Participants claimed that children should be provided an opportunity, but not obliged, to influence a choice of assistive device; goals; and when, how, and with whom rehabilitation is arranged. Participants perceived that children’s involvement in planning varied according to practices, adults’ knowhow, and the child’s age and abilities. Participation in rehabilitation planning was described as an outcome of learning.

‘I think a child can learn to participate, but they can’t participate if they haven’t been shown how to. Children may not be accustomed to being heard and thus may not engage. It’s a result of learning’ (Outi, Therapist).

Protecting children from negative encounters and problem-based discussion was important. Many parents worried about negative, problematic language being used in front of their child, for example, in rehabilitation meetings. Participants indicated that practices and policies should be reflected upon and developed from the child’s point of view.

‘You need to protect your child from that talk. They can’t be with you when you go through all the developmental delays, medical terms, and negative stuff with professionals’ (Mia, Parent).

### Fostering a fulfilling daily life for the child

*Building a participatory learning environment in everyday life* was a key practice in promoting the child’s best interests. Professionals described the importance of focussing on participation outcomes in daily life. This was initiated by measuring a child’s participation in real-life situations and through establishing participation goals. Enabling child’s participation and influencing in activities with peers and family, fostering their learning, play, enjoyment, and engagement in daily life and community, was crucial. Therapists encouraged friendship-building, peer-support, and social participation through inclusive therapy sessions, group activities, and facilitating real-life interactions with peers.

‘It’s important to enable participation in play in a way that the child can influence what the play is about. Not just being there, but really engaging’ (Teemu, Therapist).

Promoting child’s best interests included protecting children’s rights to participate through awareness and advocacy, particularly in educational settings. Modifying support structures and addressing barriers was crucial for promoting a child’s participation. Identified barriers included a lack of information, suitable leisure groups, personal assistants, scheduling difficulties, and physical and attitudinal barriers in activity environments. Important practice was to ensure that the child’s special needs are met in daily life, with guidance and encouragement from parents and professionals being essential. Collaborative planning, resource sharing, and testing facilitated learning opportunities and inclusion of rehabilitative practices in a child’s everyday routines.

‘It would be in the best interests of the child to invest a lot in their everyday environment to help parents and other people close to them; everyday life as a whole should be as rehabilitative as possible instead of the current weekly hour-long sessions with the child’ (Teija, Therapist).

Both parents and professionals underlined that new practices were required to build better collaboration, guiding, and influencing a child's daily environment. Participants noted a lack of effective tools and practices, along with confusion about responsibility for fostering collaboration and promoting change in everyday environments.

*Enhancing the child’s functioning and a meaningful daily life throughout the life course* promoted the child’s best interests by anticipating and optimising their abilities now and for the future. Participants reported that promoting a child’s best interests included discussing how to secure their functioning and wellbeing, and their happiness and quality of life now as well as in a long-term. Participants stated that rehabilitation enhanced the child’s meaningful daily life in adulthood by optimising the capabilities needed for participation, independence, employment, and accommodation.

‘Securing what can make my child happy. I think she hopes to have friends and be independent in some way in the future --. That she can do things she wants to do’ (Anne, Parent).

Prohibiting restrictions to functioning, preventing pain and surgical operations, and maintaining mobility were emphasised during adolescence. Some professionals underlined that adolescents should have an opportunity to practice self-care and independent living skills and receive support for dealing with the issues surrounding puberty, disability, physical integrity, sexuality, and identity. Special attention was needed during transition phases. Participation in society and social involvement were seen as essential components of a meaningful daily life in childhood, as well as in adulthood.

‘It's social aspects and participation that are most important. The way, how you rush in, doesn't matter so much as long as you get involved in society’ (Saara, Therapist).

Many participants emphasised that children’s communication and interaction skills should be prioritised because a fulfilling life requires social relationships and involvement in the community. Some professionals also saw language as essential for developing cognitive skills and independence.

‘Communication is the top priority – that I’ve learned. The ability to communicate is the basis of all human interaction, work, and leisure. You need to make sure that the child can speak or express themself in one way or another’ (Heljä, Paediatric Neurologist).

### Ensuring appropriate rehabilitation

The purpose of *managing a beneficial rehabilitation process* was to ensure that the child benefits from rehabilitation and their functioning improves through a motivating, goal-oriented process. All participants stressed determining goals collaboratively with parents, but many also stated that goals identified by the child are most motivating.

‘And then, of course, what that child wants, what they are motivated to do. -- You know, that they can choose too. Because that’s the best thing for rehabilitation, motivation’ (Susanna, Parent).

A beneficial rehabilitation process encompassed establishing achievable goals and realistic plans. Professionals perceived that before setting rehabilitation goals the child’s basic everyday needs and security should be safeguarded. Managing beneficial rehabilitation comprised motivating, fun, and efficient therapy sessions and exercises in suitable conditions and real-life circumstances. Parents valued a therapist’s creativity in finding a child’s areas of interest. In contrast, they considered it burdensome if their child was unmotivated to participate. Professionals indicated that sometimes it was in a child’s best interests to take a break from therapies.

Assessing disadvantages in rehabilitation was needed to prevent harm to the child and family. Parents described that rehabilitation should be arranged in such a way that it would not add burden to their daily life. They stated that currently integrating work duties, taking care of the child’s daily needs, and navigating the various rehabilitation and health services were strenuous and time consuming. Participants indicated that rehabilitation should be arranged based on needs rather than diagnosis and underlined a need for flexibility to adjust rehabilitation to changing situations and needs.‘Increasing flexibility, that would serve all families. It would reduce that everyday strain’ (Leila, Parent).

*Safeguarding equity and quality* in rehabilitation meant securing impartial access to rehabilitation and ensuring good-quality care. Some professionals worried that children with educated and resourced parents who knew what and how to demand services gained better rehabilitation and more of it. Some parents stated that you had to fight for your child’s best interests.

Varying professional practices, resources, and structures in different settings influenced rehabilitation provision, causing a risk of inequity. Promoting a child’s best interests was perceived as puzzling and unclear, lacking a common understanding. Some professionals noted that practical work was not guided by a child’s best interests due to a lack of knowledge and time resources.

Professionals expressed a need for more knowledge on child-specific and participation-based practices, along with evidence-based guidelines for promoting a child’s best interests. Multiprofessional collaboration and knowledge exchange increased the quality and success of rehabilitation. Participants described that it would be in a child’s best interests to combine services and knowledge from different disciplines. This possibility was now lacking because of policy restrictions.

‘…you should have certain basic principles (for rehabilitation according to a child’s best interests) that you can build on. And then you need individual flexibility, but those basic principles would secure equity’ (Noora, Therapist).

A few professionals emphasised *objectivity in determining a child’s best interests*. They stated that professionals who are not emotionally invested in the child’s and family’s daily life should be the ones to determine appropriate, realistic rehabilitation. When emphasising objectivity, the professionals who did not provide rehabilitation in the child’s daily life primarily based their decisions on information regarding the child’s and family’s needs.

## Discussion

This study based on constructing knowledge through social interaction (Guba and Lincoln, 1994) yielded in multifaceted descriptions of current and required practices promoting a child’s best interests in paediatric rehabilitation. The practices for promoting a child’s best interests were intertwined, anticipating and following a rehabilitation process. The findings indicate the need for child-specific practices (Coyne et al., 2016) and stronger collaboration (Coussens et al., 2022; Piškur et al., 2016). The findings emphasise the need to move from the silo-like implementation of rehabilitation and separate goals to a collective framing of rehabilitation attached to the child’s everyday life. A lack of established processes and recognised practices hindered the realisation of a child’s best interests in practice (Davies et al., 2023).

Consistent with previous studies (Costa et al., 2017; Jeglinsky et al., 2012; Teleman et al., 2021), this study found that children’s active role in family–professional collaboration is not self-evident or systematic in rehabilitation practices. The findings show the need to create a safe space for the child’s involvement in planning rehabilitation because they need protection from its problematic issues (Teleman et al., 2021) and special attention to promote participation (Coyne and Harder 2011; Davies et al., 2023). In practice, a child’s protection and participation in family–professional negotiations and planning needs careful balancing (Paul, 2007) and must take into account the child’s individual and situational needs and preferences (Coyne and Harder 2011). In line with this study’s results, Coyne et al. (2016) present that children’s participation should be viewed as an evolving process, in which the child learns to participate in supportive interaction. Sensitivity towards the child’s expressions and building trusting relationships (Coyne et al., 2016) and empowering, strength-based practices (Kronsell et al., 2021) were underlined in the results, in accordance with prior research.

This study’s findings contribute to the existing understanding, emphasising that a collaboratively constructed and comprehensive view of the child’s rehabilitation integrated in child’s and family’s everyday life is essential to safeguard their best interests. Thus, determining child’s best interests emerges as multifaceted knowledge-building, exploring and negotiation perspectives. In line with previous research, the findings underline the need to consider both child’s current and future needs (Rosenbaum and Gorter 2012) and the importance of aiming for a mutual understanding (Streuli et al., 2011) and empowering family–professional partnerships (Kronsell et al., 2021; Reeder and Morris, 2021).

The study identified that promoting child’s best interests included fostering a fulfilling daily life for the child. In rehabilitation, practices aligned with the child’s best interests promote their participation not only in rehabilitation situations but also in everyday life (Sipari et al., 2017). This involved building a participatory learning environment by adjusting factors that facilitate or hinder child’s participation (Anaby et al., 2022). Dealing with the participation barriers was challenged by unclear responsibilities, a lack of resources, and inadequate collaboration, which corresponds to previous findings (Coussens et al., 2022; Phoenix et al., 2020). In line with Anaby et al. (2022), our study findings underline the need to shift from professional-led practices towards the child’s perceptions, and a collaborative approach for facilitating the interactive processes of learning and change between the child, family, and real-life environment.

Many participants were uncertain about good practices for promoting a child’s best interests. Some of the practices were ideals rather than actually realised, day-to-day practices. Alarmingly, limited resources and a dearth of practical guidelines led to neglect of the child’s best interests (Davies et al., 2023). Although the perceived challenges in balancing between rights, principles, and professional policies (Paul, 2007), the provision and arrangement of services according to the child’s needs and rights is, however, professionals’ responsibility (Shikako-Thomas and Shevell, 2018; United Nations Human Rights Office of the High Commissioner, 1989).

The study findings present somewhat conflicting views and priorities on how to realise children’s rights to participation in healthcare encounters to promote their best interests (Davies et al., 2024; Sahlberg et al., 2020), demonstrating a need for a shared framework and practices. Because participation as a social actor in one’s everyday environment is significant for the overall development and wellbeing of a child (World Health Organisation, 2007), restrictions to participation in healthcare encounters (Coyne et al., 2016; Teleman et al., 2021) and in daily life situations (Arakelyan et al., 2019) may create a risk of exclusion from the benefits of participation. Comprehensive, collaboration-based tools such as PREP (Pathways and Resources for Engagement and Participation, Anaby et al., 2018), the Collaborative Process for Action Plans to Achieve Children’s Participation Goals (Palisano et al., 2022), and the CMAP Book (a description of Child’s Meaningful Activities and Participation, Vänskä et al., 2022) can be used to promote a child’s best interests in rehabilitation with a focus on their participation. A process-oriented practice for promoting a child’s best interests, carried out through collaboration, and based on a systemic and ecological approach, could guide the implementation of the rehabilitation process to safeguard the child’s right to participate.

### Limitations

To enhance trustworthiness, we engaged in reflexivity at every step (Elo et al., 2014). The data collection was conducted as part of the LOOK-project (https://look.metropolia.fi/in-english/). As no new categories emerged at the end of the analysis process, the researchers estimated that saturation of data had been reached. The child’s best interests’ concept is multifaceted and complex (März, 2022; Ruggiero, 2022), and the participants’ different interpretations of the study’s central concept challenged the logic of the relationship between theory, data collection, and results. Combining the parents’ and professionals’ focus groups could have yielded different results. On the other hand, separating the different participant groups may have facilitated discussions on challenging topics (Barbour 2007). Including participants from the different fields of multiprofessional rehabilitation, such as education or social services, could have broadened the perspectives in the conversations. Another limitation is that it lacked children’s views and only one father participated in the interviews.

### Implications for practice

Implementing practices aligned with a child’s best interests in rehabilitation necessitates tailoring to the child’s and family’s individual situations in family–professional collaboration. To realise a child’s best interests, healthcare professionals need child-specific practices and guidelines promoting child’s active participation. Further, there is a clear need to shift from isolated implementation and goals for rehabilitation to a collaborative approach integrated into the child’s and family’s daily life needs and possibilities. The need for evidence-driven, child-specific practices to ensure a child’s best interests is indisputable. More research on child’s best interests is particularly needed from children’s perspectives as well as from fathers’ perspectives. Also, exploring potential differences in perceptions across diverse family contexts could offer additional depth and inform more tailored approaches to supporting children’s participation. In future, guidelines for practices to promote a child’s best interests in rehabilitation should be developed in partnership between children, parents, and professionals.

## Conclusion

The study findings emphasise the importance of a shared framework for rehabilitation and construction of a participatory environment. Collectively framing a comprehensive view of a child’s fulfilling daily life is central for providing rehabilitation that promotes the child’s best interests. The findings highlighted, however, substantial challenges in family–professional collaboration aligned with the child’s best interests, enabling the child’s active participation, and addressing the individual needs of the family. The results can be used to develop guidance for promoting a child’s best interests in rehabilitation.

## Supplemental Material


Supplemental Material - Practices for promoting a child’s best interests in paediatric rehabilitation – Perspectives of professionals and parents
Supplemental Material for Practices for promoting a child’s best interests in paediatric rehabilitation – Perspectives of professionals and parents by Nea Vänskä, Salla Sipari, and Leena Haataja in Journal of Child Health Care.
