# Supplementary material for: Practices for promoting a child’s best interests in paediatric rehabilitation – Perspectives of professionals and parents
Source: J Child Health Care. 2024 Sep 28;29(4):935–50. doi: 10.1177/13674935241287880 (PMC12662835; doi:10.1177/13674935241287880)
Supplement: Supplemental Material - Practices for promoting a child’s best interests in paediatric rehabilitation – Perspectives of professionals and parents [file sj-pdf-1-chc-10.1177_13674935241287880.pdf]

Supplemental material for article “Practices for Promoting a Child’s Best Interests in Paediatric Rehabilitation – Perspectives of Professionals and Parents”

Supplementary Material 1, Table 1. Study interviews and participants’ distribution.

Interviews (n=11)	Participants (n=39)
**8 group interviews**	1. group n = 8 (4 pt, 3 ot, 1 st)*<br>2. group n = 8 (4 pt, 3 ot, 1 st)*<br>3. group n = 7 (3 pt, 3 ot, 1 st)*<br>4. group n = 3 (2 mothers, 1 father)<br>5. group n = 2 (2 mothers)<br>6. group n = 4 (4 mothers)<br>7. group n = 2 (2 paediatric neurologists)<br>8. group n = 2 (2 paediatric neurologists)
**3 individual interviews**	n=3 (paediatric neurologists)

*pt = physiotherapist, ot = occupational therapist, st = speech therapist

Supplemental material for article “Practices for Promoting a Child’s Best Interests in Paediatric Rehabilitation – Perspectives of Professionals and Parents”

Supplementary Material 2, Table 2. Subcategories, categories, and main categories.

Subcategories (46)	Categories (9)	Main categories (3)
- Identifying rehabilitation needs and preferences<br>- Multiprofessional assessment of the child’s functioning, wellbeing, and development<br>- Identifying risk factors and problems in daily life<br>- Combining experience- and evidence-based knowledge<br>- Exploring parents’ perspectives<br>- Exploring and including the child’s perspectives	**Defining an overall picture of rehabilitation needs and preferences**	**COLLECTIVE FRAMING OF CHILD-SPECIFIC REHABILITATION**
- Negotiating different perspectives<br>- Deliberation over what is in the best interests of the child<br>- Unifying goals and plans for meaningful rehabilitation in rehabilitative network<br>- Drawing up a comprehensive rehabilitation plan	**Reaching a shared understanding of the child’s overall rehabilitation**	

- Supporting parents' active roles<br>- Arranging parental support for daily tasks and coping<br>- Enabling trust and continuance in the family–professional relationship<br>- Learning to help the child together<br>- Supporting empowerment of the family<br>- Helping the child and family members deal with and accept disability<br>- Envisioning an empowering future<br>- Supporting the child's positive and realistic self-perception<br>- Focusing on strengths and solutions	**Building an empowering partnership**	
- Enabling the child's opportunities to influence<br>- Providing understandable information to the child<br>- Helping the child learn how to participate in rehabilitation planning<br>- Respecting the child's personality<br>- Protecting the child from negative encounters and problem-based discussion<br>- Developing practices from the child's point of view	**Creating a safe space for the child's involvement in rehabilitation planning**	
- Focusing on participation outcomes in daily life<br>- Supporting social involvement and friendship-building<br>- Protecting the child's participation rights in daily life<br>- Modifying supports of and barriers to participation in the environment<br>- Ensuring that the child's special needs are met in daily life<br>- Enabling the child's learning opportunities in daily life<br>- Integrating rehabilitation into daily life activities<br>- Building a rehabilitative network in the child's daily life	**Building of a participatory learning environment in everyday life**	**FOSTERING A FULFILLING DAILY LIFE FOR THE CHILD**
- Anticipating and optimising the child's functioning now and for the future<br>- Securing a good daily life in childhood and adulthood<br>- Prioritising communication and social involvement	**Enhancing child's functioning and meaningful daily life throughout the life course**	
- Ensuring a goal-directed rehabilitation process<br>- Arranging effective therapy sessions and motivating rehabilitation<br>- Flexible tailoring of rehabilitation based on needs<br>- Assessing the benefits and disadvantages of rehabilitation	**Managing beneficial rehabilitation process**	**ENSURING APPROPRIATE REHABILITATION**
- Ensuring impartial access to rehabilitation<br>- Fighting for the child's best interests<br>- Equal practices for determining the child's best interests in rehabilitation<br>- Knowledge of the child's best interests and participation-based practices<br>- Fostering multiprofessional collaboration and knowledge exchange	**Safeguarding equity and quality in rehabilitation**	
**Objectively determining the child's best interests**		
